# Transformation and Design Thinking: perspectives on sustainable change, company resilience and democratic leadership in SMEs

**DOI:** 10.1365/s42681-022-00028-x

**Published:** 2022-01-18

**Authors:** Daria Habicher, Greta Erschbamer, Harald Pechlaner, Linda Ghirardello, Maximilian Walder

**Affiliations:** Eurac Research, Bolzano, Italy

**Keywords:** Design Thinking, Transformation processes, SMEs, Company resilience, Digitalization, Democratization, Leadership, Sustainability, Socio-ecological transformation

## Abstract

This paper focuses on Design Thinking as a tool for initiating transformation processes both for the enhancement of company resilience, involving challenges connected to current trends such as digitalization and democratic leadership, as well as for the restructuring of a sustainable socio-ecological company organization. Primary findings of this explorative study show that Design Thinking is a suitable method to promote digital, democratic, and innovative business and leadership transformation, but that it is not primarily operationalized in the context of a further-reaching socio-ecological transformation towards more sustainability.

## Introduction

The complexity and especially the speed of change in society, politics, and corporate environments have dramatically increased in recent years. At a breathtaking speed, which was furthermore accelerated during the Covid-19 pandemic, several successful market players have disappeared into insignificance. Technological change and digitalization bring great benefits to businesses, but at the same time raise new political, social, and ethical challenges (European Commission 2018). New globalization trends, by some described as re-globalization (Benedikter and Kofler 2019), the demographic change of society, and climate change are **megatrends** that force companies to constantly reorganize and rethink existing structures to ensure their economic viability. More than ever, these developments challenge companies to cut their costs, improve the quality of their products or services, and find new opportunities for qualitative or quantitative growth (Reed and Luffman 1986; Agostini et al. 2020). Indeed, the cruciality of digitalization has become evident not only for large firms or innovative start-ups but also for SMEs (Fletcher and Griffiths 2020). Increased digitalization accelerates, on the other hand, collaborative and thus more democratic patterns of company organization, with employees getting increasingly involved in decision-making processes. These novelties call for new forms of leadership, to be able to integrate the young workforce as well as to develop adequate strategies to face the dynamism of market expectations (Harrison 2018).

Addressing **company resilience** seems thus to be a logical reaction to the current fast-moving economy, but also in light of evolving occupational profiles, lifestyle, and working preferences. Indeed, transformation processes in relation to advanced digitalization, company democratization, and the question of leadership seem to have become indispensable for firms and SMEs. To address and solve these kinds of challenges, Design Thinking offers a new approach by fostering change through creative and participative activities (Brown 2009). Therefore, it might serve as a useful toolset for companies to initiate transformation and to practically implement change processes. The present paper seeks to find answers to the following questions: *Is Design Thinking beneficial for the enhancement of company resilience? Does it go along with the democratization of leadership, and what role does digitalization play in this dynamic?*

In addition, the present study reaches further by considering **transformation processes** also from a **socio-ecological perspective on sustainability.** In other words, *are companies motivated to develop socio-ecologically sustainable transformation processes? And can Design Thinking be helpful in this regard?* In recent years, a paradigm shift towards sustainability has started to emerge in the fields of macroeconomics and business (Pechlaner et al. 2021). However, sustainable business transformations comprehend multilayered, irreversible, and structural change processes affecting all areas of a firm (Wunder 2017; Schneidewind 2018). Sustainable companies may moreover function as agents for wider, societal socio-ecological transformation processes (Scholl and Mewes 2015; Hiß and Nagel 2017; Habicher 2021a).

To sum up, the present paper focuses on Design Thinking as a stimulating tool to nudge transformation processes both for company resilience including the ability to adapt to current megatrends like digitalization and democratization, as well as for a sustainable restructuring of the company and, as such, for driving a systemic socio-economic change. The attention will hereby lay on SMEs, while the study adopts a qualitative- explorative research approach. Therewith, the added value of this paper lies in the combined analysis of the two different finalities that transformation processes can pursue. This interdisciplinary approach moreover enables a better understanding of the usefulness of Design Thinking not only for business scopes and company resilience but also for further-reaching socio-economic transformation by highlighting the importance of local stakeholders (SMEs) acting as drivers for a global, sustainable change.

## Literature review

To investigate the usefulness of Design Thinking as a tool to enhance SME’s company resilience—comprising digitalization, democratization, and new forms of leadership—on the one hand, and sustainable change, on the other hand, the mentioned concepts will be considered in more detail.

### Design Thinking

Design Thinking offers tools and methods to identify problems and to solve them by fostering new ideas and innovation through creative and disruptive processes of participation and collaboration (Brown 2009). For example, Scuttari et al. show that the integration of Design Thinking in operation and business management processes is a way of initiating social practices that not only help solve a specific problem but support also the adaptation of new approaches to conceiving phenomena and their interconnections differently (Scuttari et al. 2021). Volgger et al. moreover highlight the possibility of adapting design approaches not only to theories but also to practices of destination development. Design Thinking thereby explores how a transdisciplinary fusion of notions can ultimately result in a new vision (Volgger et al. 2021). Besides improving and developing destinations, instruments based on Design Thinking are considered also useful tools for firms who seek to restructure their internal processes and structures. Design Thinking might in fact enable a new and deeper understanding of problems, seeking solutions based on client experience and user-oriented perspectives. In this process, they promote integrative and transdisciplinary skills and competencies as well as a horizontal, democratic collaboration culture amongst team members (Brown et al. 2009). Moreover, Design Thinking combines isolated thoughts in creative and disruptive manners and can therefore lead to an increased openness towards radical ideas and unusual practices. Additionally, through Design Thinking, processes may be planned by following a precise procedure, but at the same time may offer a lot of room for creative and unusual ideas (Cross 2011).

Thanks to these change fostering properties and to its profoundly holistic perspective on economic, political, and social practices, Design Thinking can support transformation processes both within companies and on the societal level, fostering company resilience as well as socio-ecological sustainability (Brown et al. 2009; Sommer and Welzer 2017; Jonas et al. 2015). In fact, applied examples show how Design Thinking fosters future-oriented strategic planning for enhanced innovation and company resilience (Shamiyeh 2010; Knight et al. 2020) and how its effectivity rises especially if constituted as an integral part of an organizational culture based on learning processes, collaboration, risk-taking, and emotional experiences and empathy (Elsbach and Stigliani 2018). Similarly, Design Thinking can foster transformations towards sustainability because of its user-centered and iterative problem-solving approach (Buhl et al. 2019). In the ideal case, companies can show by example that the existing, user-oriented design approaches can be expanded to a society-oriented approach (Jonas et al. 2015).

Based on these considerations, the present study investigates the usage of Design Thinking tools in SMEs and their main motivations and goals for enhancing company resilience, the role of digitalization, leadership styles and democratization and sustainable change as well as their possible role as agents of a wider societal transformation towards sustainability.

### Company resilience

The concept of resilience refers generally to a good performance considering adversities and includes also an anticipating capacity to avoid, adapt to and grow from crises and shocks. Research on company resilience examines however various aspects of firms and their performance: employee-related capabilities, business processes, and organizational procedures. SMEs are generally considered to be rather poorly prepared for crises such as recessions, environmental disasters, or other disasters (Annarelli and Nonino 2016; Battisti and Deakins 2017; Wishart 2018).

For the purpose of this paper, a resilient company is conceived as a firm or organization, which is strongly adaptive, agile, and flexible. Without focusing on the recent Covid-19 pandemic, *company resilience* refers thus to the more general adaptability of a firm to respond to current challenges and adversities. Current global challenges, as mentioned in the introduction, comprise for many SMEs, among others, the three interdependent aspects of digitalization, democratization, and leadership, which will be briefly discussed in what follows.

#### Digitalization

Technological and digital solutions, such as platforms, distribution channels, and business models, serve as means to increase productivity, efficiency (European Commission 2018), and profitability. Indeed, digitalization processes have a large impact on all types of organizations and businesses, since “*everyone is affected by ‘digital’*” (Bican and Brem 2020, p. 9). However, in the current digital age, changing business environments represent great challenges, especially for SMEs. Innovation pressures and business change activities push in fact towards the digital transformation of most firms, although especially SMEs, low margin, and commodity businesses face challenges to keep up with the rapidly growing speed of digital development (Bican and Brem 2020). In fact, “*fast product cycles originating from changing customer needs”* (Bolte et al. 2018, p. 1) have accelerated and require agile production, flexible development cycles, and an up-to-date company management culture. However, research has shown that especially in SMEs expectations and knowledge about digitalization strategies are rather low (ibid).

Generally speaking, the digital dimension plays an important role when it comes to “*contributing towards the goals of the United Nations Sustainable Development Goals, where economic and environmental issues are at the heart of solving the challenges of the future*” (Bican and Brem 2020, p. 11). In fact, digital solutions are supposed to facilitate sustainability and to have a positive impact on the planet despite their well-known, controversial rebound effects (Kopp and Lange 2019). For instance, digitalization may contribute to protecting biodiversity thanks to innovations coming from the fields of artificial intelligence or the internet of things that surely foster smart and data-based policy implementations (Hedberg and Sipka 2020). Moreover, the potential of digitalization to drive social transformation has similarly been highlighted (Lange and Santarius 2020).

#### Democratization

Together with the unprecedented pressure towards the digital realm, democratization trends in firms and organizations have been highlighted in recent social sciences (Dörre 2015; Kühl 2015; Borsch and Borsch 2019; Herzog 2019; Habicher 2021b). In fact, concomitant with digitalization and enhanced collaboration tools and software, hierarchical decision-making is increasingly being replaced by shared approaches of organizational management (Hinterhuber and Krauthammer 2015; Harrison 2018; Borsch and Borsch 2019).

In this context, Corbett and Spinello (2020) argue that a possible way of reinforcing democratic structures in firms and thus also in SMEs is based on connectivism. Thereby, “*connectivism describes the nexus between human learning and the ubiquitous access to knowledge enabled by the current technological environment”* (p. 1). Indeed, the possibilities of continuous self-learning and skill acquisition through the internet increasingly recontextualize the relationship between individuals both in managerial and non-managerial positions. For this reason, networking has become central and even more important, changing habits and organizational practices in many firms. The idea of connectivism comprehends moreover *“open communication, increased engagement, distributed knowledge, and collaboration*” (p. 7)—aspects which are believed to foster profound democratization in firms. The emphasis on collaboration in fact highlights the importance of interpersonal knowledge-generating processes between individuals of various levels and departments, utterly reinforcing the democratic approach of organizational structures. Networks and technology could therefore represent a means of generating a new form of *“Connectivist Leadership”,* consisting of shared processes and structures in today’s digitalized world*.* Substituting the traditional paradigm of follower-leader with *“a collective and connected network-forming process, it challenges leading through a singular source of authority, power, control, or any form of hierarchy”* (Corbett and Spinello 2020, p. 8).

In this sense, digitalization opens up a space for democratization within SMEs and fosters ideas of team-based management. In sum, the recent technological tools that increasingly enable personal knowledge acquisition through open-source instruments, online professional training, and digital education, provoke a new possibility for the consolidation of a profoundly non-hierarchical form of company organization. In the era of rapid technological progress, where knowledge and skill are accessible, decentralized, and diffused, the notion of structural and organizational democratization processes might furthermore provide an opportunity for future management literacy.

#### Leadership

The developments between digitalization and democratization give rise to the question about leadership and how it needs to be organized in the digital era. First, to accelerate digital transformation within both large firms and medium and small-sized companies, Sow (2018) finds that solid technical knowledge and willingness to change are characteristics required by successful leaders. Pragmatically speaking, concrete change strategies and a critical understanding of the process, along with patience to withstand challenges and disruptions during the implementation phase, are moreover useful to enhance the effectiveness of digital transformation processes. Similarly, Bolte et al. (2018) plead for a new type of leadership that is coherent with the technological advancements of the last years. “*Leadership 4.0*,” as they call it, needs to be agile, in order to respond to faster business cycles and achieve higher satisfaction among customers. “*Openness, transparency as well as a lived error culture to keep the velocity high and stay flexible to drive innovations*”, is thereby considered fundamental (p. 2). On the one hand, leaders of SMEs broadly speaking can meet these requirements well. Because of their small size compared to big companies, the relatively short paths, and the often personal relations they score in speed, flexibility, and adaptability. On the other hand, they are forced to stay always in a niche and need to be of very high quality to remain competitive (Montanari and Kocollari 2020).

Moreover, Ferdig (2019) goes further by proposing a generative and innovative engagement approach both in the world of business and beyond*.* Her definition of a leader regards a person “*who takes responsibility for understanding and generating workable solutions WITH others for the ordinary and overwhelming challenges we encounter day-to-day”* (p. 2). This emphasis on responsibility on the one hand, and the collective dimension of problem-solving on the other hand, represents a democratic approach to leadership that is currently emerging both in theory as well as in practice.

However, business transformation is not the only challenge firms are facing at present. It is necessary to continuously evolve leadership styles coherent with the current entrepreneurial *and* global economic setting (Nguyen et al. 2021). For instance, ethical and moral values are in becoming important leadership qualities especially for what concerns a sustainable transformation. Ethical leadership is in fact considered to foster cohesion within companies and overall business performance (Ko et al. 2017). Concordantly, leadership strategies increasingly shift towards long-term value creation, instead of focusing primarily on short-term gains. For instance, Tidemann et al. (2013) state that leaders require “*an evolved type of consciousness, with an appropriate skill set derived from this consciousness*" (p. 24). Future leaders need thus a continuity-oriented, context-aware, open-minded, and morally courageous mindset that enables them to navigate their firm towards a sustainable business model. Additionally, a creative and interconnected leadership philosophy fosters sustainable value chain transformation for large-scale social impact (Tidemann et al. 2013; Yukl 2013).

### Socio-ecological transformation for sustainable change

Sustainability has become one of the main concerns in face of the current climate crisis, the loss of biodiversity, and the deterioration of the environment, showing that human impact has already reached far beyond the bearable limits of the planet (UN 2015). In this context, it is known that especially the negative effects of the advanced market economy and “business as usual”, contribute to the ecological crisis through their massive CO2 emissions, waste production, and the release of chemical substances (Meadows et al. 1972). Because of this, traditional business models are being revisited in a sustainable optic (Wunder 2017). Sustainability is thereby framed as an ethical and moral issue representing the main goals in restructuring and reshaping firm organization and processes (Tidemann et al. 2013).

Business transformations aiming at the limitation of a firm’s negative impact on the planet are therefore another aspect of transformation processes which include sustainable business models and strategies such as CO2- or climate neutrality, circular economy approaches, short supply chains and regional business cycles, eco-friendly products, and materials, sustainable investments, etc. (Murray et al. 2017; Dentschev et al. 2018; Cantele and Truzzi 2020).

However, a sustainable business transformation needs to be understood as comprehensive management-, leadership, and decision-making system that affects all tangible and intangible areas of the company: standards, processes, structures and systems, and ultimately corporate culture (Schulz 2017). In a much broader sense, sustainable transformation can be thus defined as a large-scale, multilayered change process that requires irreversible modifications and provokes a so-called paradigm shift. This comprises structural changes including underlying cultural, moral, and value patterns, as well as production and consumption behavior, technology, and infrastructures (Schneidewind 2018). Firms have, in this regard, the potential of fostering a more sustainable market economy, and becoming decisive agents for driving systemic socio-ecological transformation processes towards sustainability (Scholl and Mewes 2015; Habicher 2021a).

As this brief literature review shows, business transformations aiming at consolidating company resilience or at fostering socio-ecological sustainability are both needed in light of current megatrends affecting individuals, companies, and entire societies and economies. Because of its innovative, creative, and disruptive features, Design Thinking can represent an ideal toolkit to accelerate or initiative such transformations. The facilitation of both digitalized and democratic company structures, an open, perspicacious, and conscious leadership as well as ethically responsible and socio-ecologically sustainable business models may thus be achieved through Design Thinking. The empirical results concerning the effective usage of Design Thinking for the above-mentioned purposes will be presented in the next chapters.

## Methodology

To answer the research question about the role of Design Thinking in fostering company resilience and socio-ecological sustainability, a qualitative and explorative research approach was chosen. The interdisciplinary and multi-thematic approach of this paper emerged as a result of preliminary test phases of the interview guidelines and was adopted to gain multifaceted insights into the manifold realities of SMEs confronted with various challenges. In a second step, semi-structured interviews (Flick 2016) were conducted virtually between August and December 2020 with business consultants who accompany SMEs in their business transformation using Design Thinking tools. Comprehensively, twelve consultants were interviewed, among which five women and seven men from three different countries. Among those, four consultants worked predominantly in Italy, three interviewees in Austria, and the remaining eight in Germany. Each interview was however conducted in the respondent's preferred language. The interviewees were chosen following preliminary internet research while subsequent respondents were added through snowball sampling.

The choice of business consultants can be motivated because of their expertise with the practical implementation of innovative methods such as Design Thinking for and with businesses. Consultants, therefore, need a deep understanding of both the tools, strategies, and theories and gain an enhanced overview of the current challenges, strengths, and motivations of the many SMEs they advise and accompany through various transformation processes. All interviewed consultants actively use Design Thinking in their business consultancy for SMEs. Their perception and insights of the current situation of SMEs concerning digitalization, democratic leadership, and sustainable change can thus provide precious results.

Finally, for the analysis of the interviews, the software GABEK^®^-WinRelan (Holistic Processing of Linguistic Complexity), a computer-assisted tool that allows to code the transcripts on a keyword basis, was used. The outcome consists of association graphs, which are visual representations of the respondents' statements. These graphs can be seen as semantic networks showing how many times a keyword is mentioned within the same conceptual unit, where the central, grey term represents the key term (Zelger 1994; Buber and Zelger 2000). The following section will highlight the results of the analyzed interviews. The section is structured along with the most important keywords which were used in the analysis with GABEK^®^-WinRelan.

## Results of an exploratory study

### Motivations for business transformation and main challenges for companies

**Fig.1:**
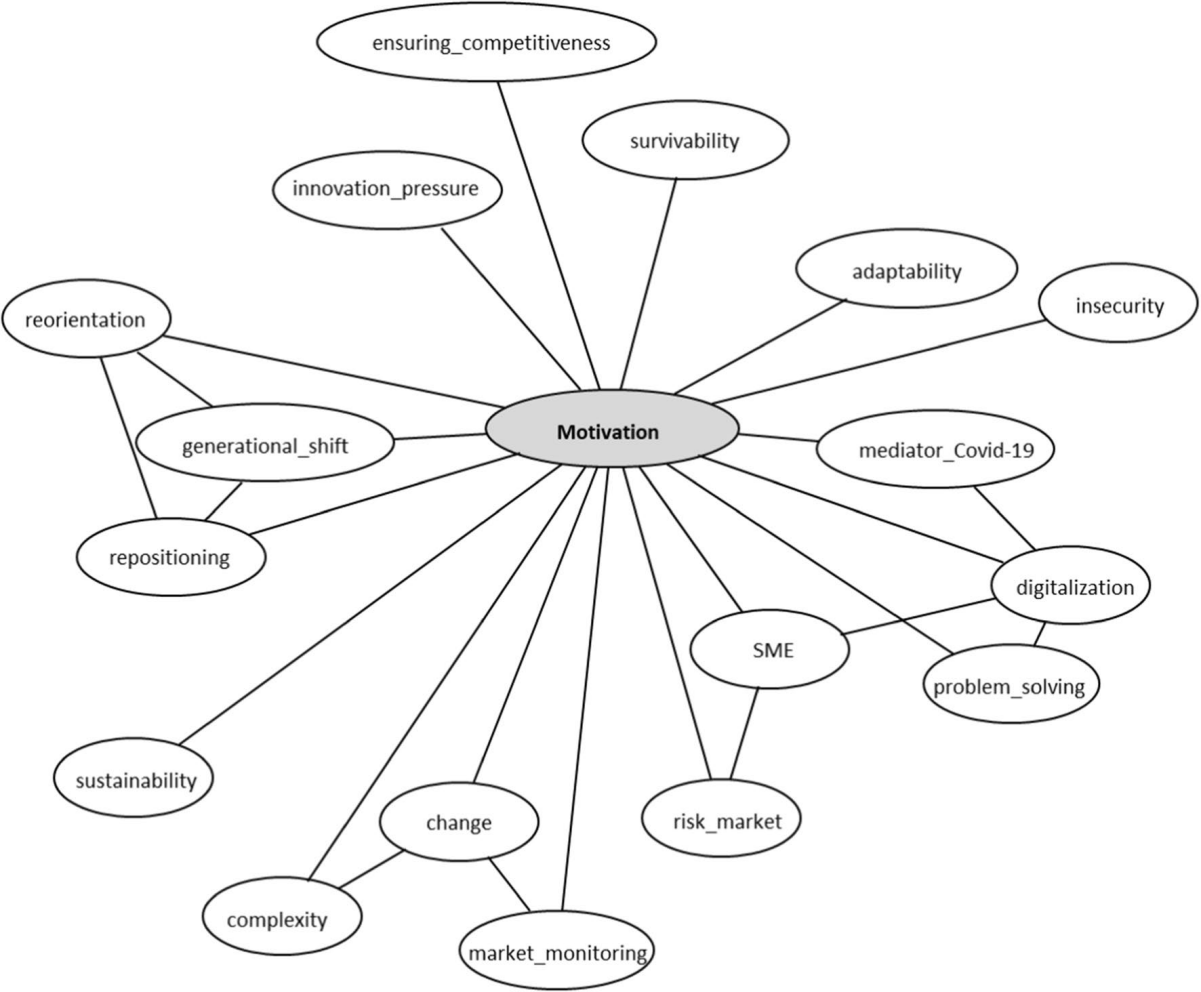
Motivations for business transformation (own elaboration)

Interview respondents were asked about the main motivations of their clients to carry out or nudge transformation processes (see Fig. 1). In this regard, the digitalization of company structures was named to be a central desire for businesses, as increased digitalization is considered to have a positive effect on problem-solving for the mitigation of market risks and the adaptation to changes and complexity, especially for what concerns family-led SMEs. Moreover, some companies seem to undergo transformation processes because of a general business re-orientation due to a generational change in management positions, while others convey the impression of recognizing the need to become more sustainable. However, sustainability was here conceived both as the ability to withstand the pressure of innovation and to ensure competitiveness, as well as in a broader sense, contributing to a more ecologically sustainable world.

Another question dealt with challenges companies seek advice for. It emerged that the reasons why firms consult business strategists are diverse but primarily related to specific difficulties related to future challenges. One consultant explained: “*It's always about setting up for the future, so how do we set ourselves up for the future?*" In this regard, the launch of new products and services on the market, as well as innovative business models, agile structures and processes, as well as company organization were evoked. Other challenges resulted to be related to the topic of communication, either towards clients or internally towards employees. In this context, digitalization was often seen as a solution to increase efficiency and to secure a steady market position. In some other cases, companies were aware of their internal problems but lacked solutions. Digitalization was thereby considered useful for the transformation of internal processes and structures.

### Design Thinking, structural conditions, and types of leadership

Another aspect explored during the interviews concerned framework conditions for Design Thinking to be applied in transformation processes and the usefulness of Design Thinking tools to initiate or foster change in SMEs. The structural conditions and prerequisites that need to be in place in an organization seem to be first and foremost openness on the side of the employees, but also on the side of the firm’s managing and executive board. It was stressed that although, theoretically speaking, Design Thinking is applicable for all kinds of businesses, regardless of their size, orientation, and sector, the practical implementation of Design Thinking differs. In general, it emerged that for Design Thinking to bear fruits, all parties concerned must be open and curious to change, must accept the fact that change is necessary, and be open for criticism. In other words, or as an interviewee put it: "*I simply believe* [that it is important to] *have a basic positive attitude*”.

**Fig. 2:**
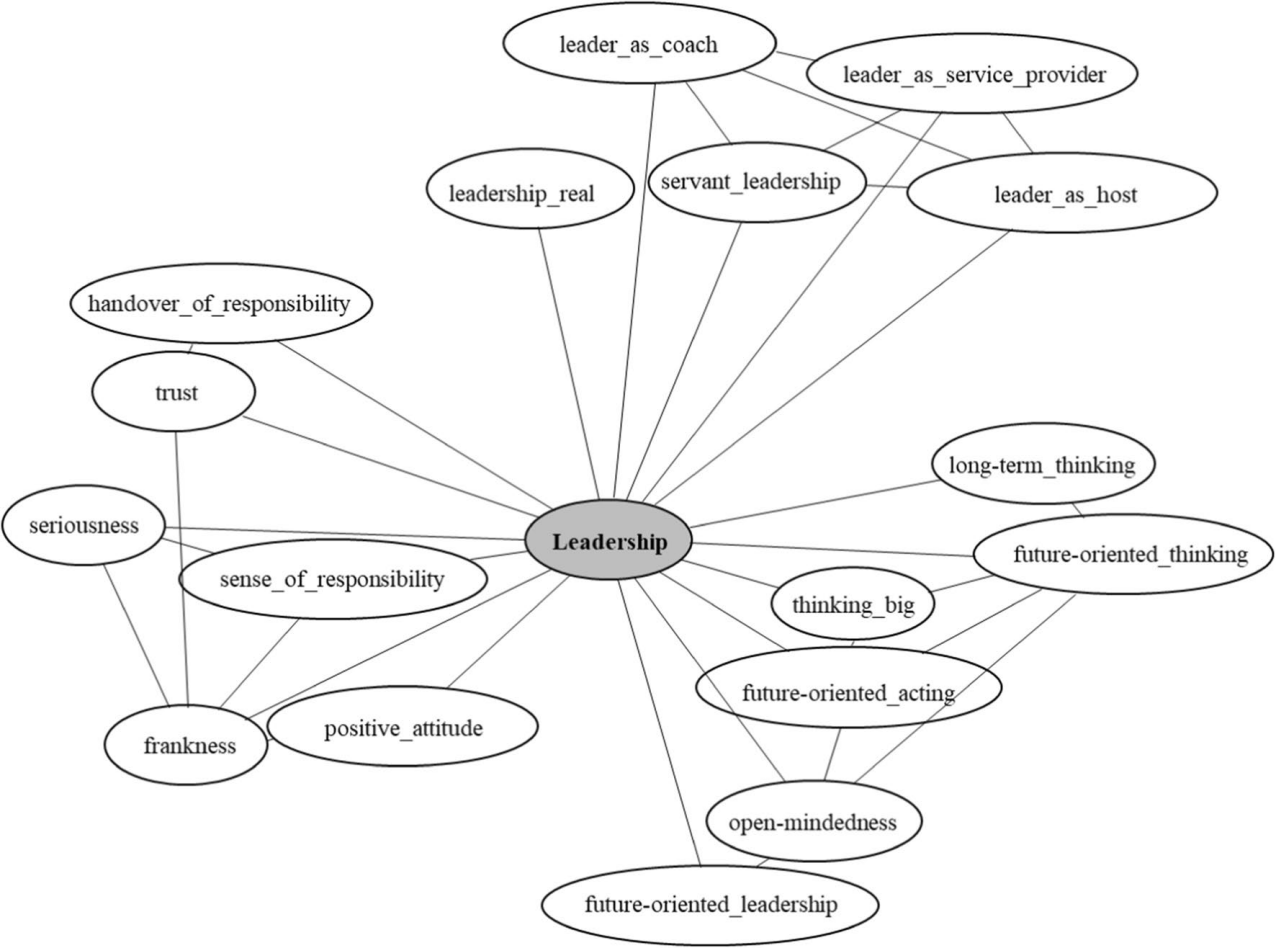
Relation between leadership and design thinking (own elaboration)

Design Thinking was furthermore described as a very horizontal and participative approach, which means that employers and employees work at eye level towards transformation and innovation. This can nevertheless only be initiated at the management level. To be successful, Design Thinking requires therefore a special kind of leadership. Company leaders must first and foremost be committed and convinced of the method itself and have a considerable amount of trust in the involved employees. Especially for SMEs, which in our context were mostly family businesses, this might cause initial irritations, as “*family fathers who have always determined everything themselves”* must hand over decision-making power to others. This of course is not exclusively true for *“family fathers”* at the top of businesses but is meant to show, that for SMEs it might be challenging to break long-lasting hierarchies in traditionally structured environments. In this context, the ideal leader was described to behave more like a coach, a moderator, a servant, or a provider (see Fig. 2). A leader should therefore not let her/his employees do the work but should lead by example and work together with all the rest, even work for their employees. These statements reflect the democratic horizontality of organization Design Thinking demands as an approach. In fact, it can only be successful if everyone in the company meets on a plain playing field and gets involved in democratic discussions and the exchange of ideas. This sort of participative leadership was described by some respondents as *“real leadership”*. It also was stressed that for what concerns SMEs, it is easier to involve most of the personnel in the transformation process.

Moreover, Design Thinking seems not to be suited for short-sighted goals, for instance, to boost company statistics before a merger or sale. The ideal leadership is considered to be future-oriented and long-term, which demands a stronger commitment to the company and its ambitions both by leaders and employees. To put it in other words "*transformation is no longer possible if someone no longer believes in the future*", a respondent claimed. Real transformation, therefore, needs time and space to evolve and cannot be rushed. Most respondents agreed that one must work for the future and not just "*administer the present*”.

### Digitalization and Design Thinking

Digitalization was mentioned to be one of the most important drivers for transformation. The respondents highlighted that “*a lot has been done in the last 5 years, but there is still a lot of room for improvement*”. This was nevertheless sped up significantly in times of Covid-19. The interviewees underlined that especially SMEs need to catch up on digitalization to remain competitive and relevant on the market. In fact, respondents stated that in the study area (Northern Italy, Germany, and Austria) there is a lot of catching up to do. Some even described digitalization as one of the big challenges for today’s companies, because a lot of businesses and especially SMEs missed following the trend. Now it is their challenge to close the gap to already digitalized companies, although this step seems to be overwhelming for some company leaders. Nevertheless, the need to tackle the challenge of digitalization was highlighted several times, since “*digitization is here to stay and if you are not there, you have a problem*”.

Digitalization has increasingly become a central reason for the initiation of organizational business changes as well as a reason for seeking advice from consultants. Respondents stated that the definition of what digitalization of businesses means and what companies expect from it differs widely. Some “*problems that come from companies are very concrete*”, for instance, the creation of an online shop or a website, while others seemed to aspire to change their whole work processes, business models, or company culture. Some interviewees said that companies want more transparent, standardized, and controlled processes. Digitalization is also strongly connected to an increase in efficiency as companies gain time that can furthermore be invested in e.g. customer support or service. At the same time, it emerged that customer relations can be rendered more efficient through digital tools: “*I don't always have to go to the customer, I can also do it from home*” was an emblematic sentence for the benefits of digitalization. In fact, automized processes not only speed up transactions but also simplify and structure them, the respondents stated. These results show that digital aids are an important and simple first step for SMEs to initiate a digitalization process demanding a certain level of digital literacy. But once employees and employers get comfortable with new digitalized processes and technological tools, to use them in their day-to-day work, the company benefits from them. As an interviewee stated: "*it can help, as I said, to make things easier, faster, more efficient*”.

**Fig. 3:**
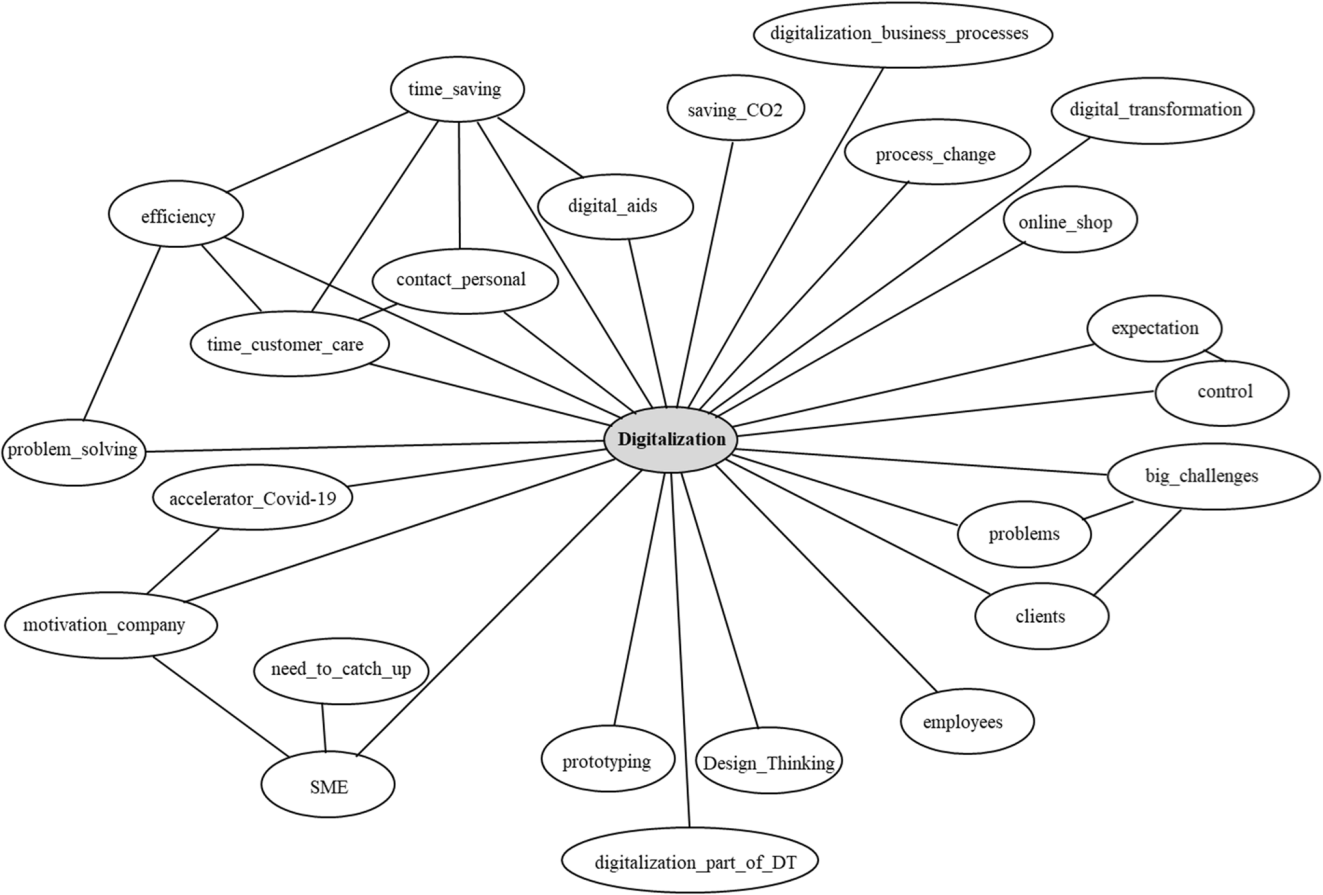
Relations between digitalization and Design Thinking (own elaboration)

When asked about the relation between digitalization and Design Thinking, respondents overwhelmingly stated that the two are strongly interconnected. This is not to say that Design Thinking is a driver for digitalization, but the two concepts are easily combinable and work well together. Some claimed that nowadays transformation must include digitalization strategies, or even considered digitalization as such as a transformation process. Others conceived digitalization merely as a small part of the whole transformation process stimulated by Design Thinking. In any way, digitalization is, more than ever, a central challenge companies must tackle, and Design Thinking seems beneficial in this regard (Fig. 3).

### Socio-ecological change and Design Thinking

One of the core questions of this study was to find out if Design Thinking processes promote far-reaching, societal transformations in the sense of socio-ecological change towards sustainability. In this regard, respondents stated that for companies, societal changes are mostly only a secondary goal while the primary objective was maintaining or achieving company competitiveness. Interviewees explained that if there was greater awareness of this potential, then maybe companies would also think beyond their office walls.

However, consultants affirmed that there are also companies actively seeking socio-ecological transformation. Especially for socially responsible firms, Design Thinking resulted to be very useful to encourage further socio-ecological transformation. Indeed, Design Thinking processes were considered as helpful tools for the formulation of ideas for change and forwarding causes on societal matters. Some interviewees underlined that societal change must begin at a small level, and then expand to impact larger parts of society. One interviewee brought it to the point by saying that “*this means that if I start to transform myself, to change, then perhaps my environment will also change, then my children will also change, and in the long term they will also take this mindset with them and thereby perhaps actually create an impact on society”*. This and other statements reinforced the idea that companies that undergo internal transformation processes for a social goal such as treating employees better or producing in a more eco-friendly manner initiate also societal transformations through the spillover effect of their own business changes. To sum up, the consensus of our interviewees showed that Design Thinking does not promote societal transformation per se. It was rather considered as a tool to help companies with the promotion of their core values and goals, which can but do not have to include social or ecological causes.

## Discussion

The explorative study shows that Design Thinking is indeed a welcome tool to initiate or foster change in SMEs. The transformation processes are hereby mainly motivated by enhancing innovation and agility to withstand estimated market risks. In other words, resilience and adequate preparation for future developments and disruptions seem to be the main motor for initiating or furthering business change. Another motivation to undergo a business transformation lays in the need for re-orientation due to a generational change in management positions. A third motivation mentioned by the consultants is the increasing need to become more sustainable. In this context, this motivation was conceived more as the ability to withstand the pressure of innovation and to ensure competitiveness, and to a lesser degree as contributing to a more ecologically sustainable world. It might seem surprising that firms did only scarcely consider long-term ecological and societal transformation processes as primary goals, as economic viability surely depends on ecological and social factors as well. On the other hand, SMEs often do not have the financial means or the knowledge to deal with ecological and social issues when the survival of their own business is at stake (Habicher 2019).

The integration of new instruments, such as Design Thinking however shows that the qualities of openness and the willingness to try new methods are considered important elements for corporate development. In this context, leadership based on horizontal approaches seems to match the participative and collaborative nature of Design Thinking very well. Indeed, the perception of leaders as coaches, moderators, servants, or providers exemplifies the trend of increased democratization in company culture. Openness was mentioned as a very important feature of the future leader, just as Bolte et al. (2018) mentioned in their study, citing moreover also qualities such as transparency and a lived error culture for enhanced agility, flexibility, and innovative potential. Connectivist leadership, as emphasized by Corbett and Spinello (2020), furthermore could indeed be a very good approach for firms that aspire to improve the intersection of democratization and digitalization within their structures. Design Thinking is hereby a great approach to foster egalitarian participation and to enable knowledge exchange (Jonas et al. 2015; Sommer and Welzer 2017; Brown 2009), as Design Thinking tools are also available digitally. The Design Thinking approach might therefore be challenging for some businesses, especially family-owned SMEs, with long-lasting hierarchies and traditionally structured environments, which must be rethought.

After the Covid-19 outbreak, the need for digitalization has become an even more central reason for the initiation of organizational business changes as well as a reason for seeking advice from consultants. Overall, digitalization seems to be both a challenge and a solution to many firms, although in this case, the sector of operation plays a significant role. Technology-affine companies surely have a greater advancement and potential for digital solutions and digitalized internal processes, while other, more traditionally organized businesses outside the tech sector, lag. Also, the generational composition in the companies’ leadership plays an important role in this regard, as solid technical knowledge, and willingness to change, as also emphasized by Sow (2018), are typical features of the new generations. Our interviews also showed that digitalization and Design Thinking are easily combinable and work well together. Consultants underlined that every business transformation process nowadays should also involve a digital transformation.

Besides promoting business transformation, Design Thinking promotes also far-reaching societal transformations. Within companies, the aim to reach societal changes by business transformation is mostly only a secondary goal. Nonetheless, consultants are convinced that one should encourage the usage of Design Thinking to boost a socio-ecological transformation.

The issue of sustainability which will probably gain increased importance for companies in the years to come (Grothe 2012; Wunder 2017; Pechlaner et al. 2021), was not sufficiently radiated in the consciousness of most consultants and the firms they advise. Indeed, while economic sustainability and resilience were high at stake for most firms, more holistic approaches to the idea of socio-ecological sustainability were in large part lacking. According to the interviewed business consultants, only firms that were already ecologically and socially oriented and committed to these goals seemed to aspire to further transformations towards sustainable business models. However, the idea of and to some extent the need for a far-reaching socio-ecological transformation seems to have not reached most other companies.

To be better prepared for future challenges regarding sustainable economic processes, firms and their leaders would need to adopt mindful features such as related to understanding and being aware of the current context and megatrends, developing a socio-ecological consciousness, and thinking in long-term and continuous terms, just as Tidemann et al. (2013) mentioned in relation to sustainable business models, alongside creative and networked approaches to enhance collective wellbeing.

## Conclusions, limitations, and further outlook

The current uncertain and fast-changing times require companies to adapt quickly and constantly to maintain their market efficiency, amplifying and increasing pressure to be adaptable, innovative, and agile, especially for what concerns SMEs. In this context, the use of Design Thinking as a tool resulted to be useful for initiating innovation cycles and for fostering digitalization processes. It moreover seemed to stand in strong connection with democratic and participatory leadership approaches and networked knowledge sharing. Enhanced digitalization and the possibility of digital learning may furthermore contribute to the establishment of greater participative and horizontal collaboration patterns within SMEs. However, socio-ecological transformation and the restructuring of sustainable production and distribution processes were, according to business consultants, not the primary goals of most firms, while Design Thinking played only a marginal role for this purpose. The SMEs’ main goals remained indeed rather traditional objectives such as economic viability, business agility, and company resilience.

These first conclusions are far from being exhaustive; in fact, differences in the constitution of firms might play a significant role when it comes to transformation, digitalization, and innovative leadership processes. It would thus be interesting to investigate if the size of SMEs and their number of employees, on the one hand, and the ownership style—such as traditional family businesses versus cooperatives or start-ups –, on the other hand, to provide different results. Also, and most evidently, sector-specific differences should be investigated further, especially to find out in how far issues such as digital innovation, and/or socio-ecological responsibility are aspired goals or rather unwanted challenges. Moreover, interviews with firm representatives would provide deeper insights into the topic and peculiar business strategies, while a larger data sample would allow a more complete picture. Additionally, a more detailed analysis of the impact of Covid-19 both on company resilience as well as on the motivations of SMEs concerning transformation processes—rather oriented towards business-enhancing scopes or rather towards socio-ecological purposes—would be interesting to highlight. Finally, cross-cultural investigations could furtherly provide more detailed and generalizable results and insights into the discussed topics. Nonetheless, the adopted explorative approach enabled first insights into the motivations for business transformation, their correlated challenges, tools, and organizational processes that firms face in the fast-changing economy of today.
